# Design of an efficient medium for heterologous protein production in *Yarrowia lipolytica: *case of human interferon alpha 2b

**DOI:** 10.1186/1475-2859-10-38

**Published:** 2011-05-20

**Authors:** Najla Gasmi, Atef Ayed, Jean-Marc Nicaud, Héla Kallel

**Affiliations:** 1 Unité de Biofermentation, Institut Pasteur Tunis, 13, place Pasteur. BP 74. 1002, Tunis, Tunisie; 2 INRA, UMR1319 Micalis, Domaine de Vilvert, F-78352 Jouy-en-Josas, France; 3 CNRS, UMR1319 Micalis, Domaine de Vilvert, F-78352 Jouy-en-Josas, France

## Abstract

**Background:**

The non conventional yeast *Yarrowia lipolytica *has aroused a strong industrial interest for heterologous protein production. However most of the studies describing recombinant protein production by this yeast rely on the use of complex media, such media are not convenient for large scale production particularly for products intended for pharmaceutical applications. In addition medium composition can also affect the production yield. Hence it is necessary to design an efficient medium for therapeutic protein expression by this host.

**Results:**

Five different media, including four minimal media and a complex medium, were assessed in shake flasks for the production of human interferon alpha 2b (hIFN α2b) by *Y. lipolytica *under the control of POX2 promoter inducible with oleic acid. The chemically defined medium SM4 formulated by Invitrogen for *Pichia pastoris *growth was the most suitable. Using statistical experimental design this medium was further optimized. The selected minimal medium consisting in SM4 supplemented with 10 mg/l FeCl_3_, 1 g/l glutamate, 5 ml/l PTM1 (*Pichia *Trace Metals) solution and a vitamin solution composed of myo-inositol, thiamin and biotin was called GNY medium. Compared to shake flask, bioreactor culture in GNY medium resulted in 416-fold increase of hIFN α2b production and 2-fold increase of the biological activity.

Furthermore, SM4 enrichment with 5 ml/l PTM1 solution contributed to protect hIFN α2b against the degradation by the 28 kDa protease identified by zymography gel in culture supernatant. The screening of the inhibitory effect of the trace elements present in PTM1 solution on the activity of this protease was achieved using a Box-Behnken design. Statistical data analysis showed that FeCl_3 _and MnSO_4 _had the most inhibitory effect.

**Conclusion:**

We have designed an efficient medium for large scale production of heterologous proteins by *Y. lipolytica*. The optimized medium GNY is suitable for the production of hIFN α2b with the advantage that no complex nitrogen sources with non-defined composition were required.

## Background

The production level of heterologous proteins greatly depends on the characteristics of the host cell, the recombinant protein to be expressed, the promoter used and most importantly on the composition of the medium, showing that production can be limited at any level.

*Yarrowia lipolytica *is a dimorphic ascomycete that naturally secretes several enzymes. In recent years it has attracted the attention of researchers as a model organism in dimorphism and secretion pathway studies [1,2]. Furthermore, developments in genetic engineering and molecular biology make the non conventional yeast *Y. lipolytica *as one of the most promising hosts for efficient heterologous protein expression [3-5].

Although extensive data on *Y. lipolytica *cultivation was reported in the literature, these reports mostly describe the production of citric acid, single cell proteins or cognate proteins like lipases, and may not be always fully adapted to recombinant protein production [5-7]. Furthermore, studies on the expression of heterologous gene in this yeast rely on the use of complex media and shake-flask cultures. Nevertheless this kind of media shows numerous short-comings such as a non defined composition, a high batch-to-batch variability and a high cost. On the other hand, the majority of publications in the field of recombinant proteins production by *Y. lipolytica *report the use of the constitutive promoter hp4d [8] which can be problematic when the product being expressed is toxic to the host [4] or the use of XPR2 promoter which requires high levels of peptones in the culture medium for its full induction [9]. However the use of non-defined ingredients such as peptones is not suitable to industrial processes; for these reasons it is not only important to maximize the yield of the heterologous protein but it is essential to obtain a consistent product under the most controlled culture conditions [10].

Medium composition for *Y. lipolytica *has not been extensively studied compared to heterologous production by the yeast *Pichia pastoris*. Thus careful consideration of medium selection is advisable when optimizing the production of heterologous proteins particularly for pharmaceutical application, owing to its critical impact on the economy and the feasibility of the process.

In addition to conventional methods based on single factor variation used for medium optimization, statistical experimental design methodology was developed and applied for the design of new media or the screening of nutrient supplements. It is an efficient tool to identify interactions between the parameters tested, resulting therefore in a great reduction of time and cost [11,12].

We described in a previous work human interferon α2b production in *Y. lipolytica *under the control of a strong inducible promoter acyl-co-enzyme A oxidase (POX2), repressed by glucose and glycerol, and induced by fatty acids and alcanes. We showed that low expression of foreign genes in *Y. lipolytica *could be attributed to several factors: the genetic design of the construct such as codon bias optimization, the use of an appropriate signal peptide and an adequate translation initiation codon environment [13]. However, besides these factors, medium composition could also affect the yield of recombinant proteins production. It was reported that the productivity of lipase by *Y. lipolytica *is affected by the presence of tryptone in the culture medium and the presence of inducers [6,12,14]. In the current study, we first assessed the effect of four different minimal media as well as organic nitrogen substrates on cell growth and human interferon alpha 2b (hIFN α2b) production in a recombinant strain of *Y. lipolytica*. The selected medium was then further optimized using statistical experimental design approach. Cell growth and hIFN α2b production in a 5-l bioreactor in the optimal medium using oleic acid as a carbon source and inducer, were then described.

## Results and Discussion

To produce hIFN α2b in *Y. lipolytica *and to target its production into the culture medium, we had previously expressed this gene under the control of POX2 promoter inducible by oleic acid. Different sequences of the signal secretion signal of *Y. lipolytica *extracellular lipase encoded by the LIP2 gene were tested [13]. Best results were obtained with the strain JMY1852 expressing hIFN α2b with the preLIP2 signal peptide.

For media optimization studies, we first isolated a prototroph derivative of JMY1652 by its transformation with a fragment carrying the *LEU2 *gene as described in the Material and Methods section giving rise to the strain JMY1852p which can be grown in minimal medium.

### Selection of an appropriate synthetic medium

#### Classical media

**Figure 1:**
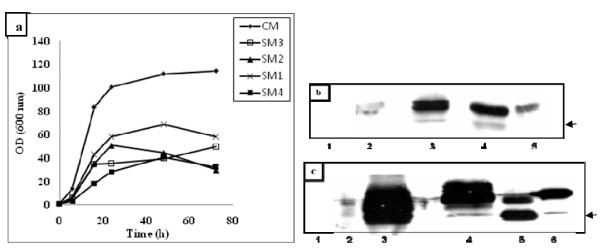
**Cell growth and hIFN α2b production profiles in different media culture**. Time course of cell growth (a). Western blot analysis under reducing conditions using monoclonal antibody (b) and polyclonal antibody (c) directed towards hIFN α2b. Lane 1, SM3; lane 2, SM2; lane 3, CM; lane 4, SM4, lane 5, SM1 and lane 6 hIFN α2b standard. The arrows indicate protein degradation. Cultures were carried in duplicate in shake flasks at 28°C and 180 rpm. For clarity reasons error bars are not shown.

Besides classical rich medium like YPD, several defined media have been previously used for *Y. lipolytica *cultivation such as the medium SM1 formulated by Olssen and Johnson [15], the medium (SM2) proposed by Gordilo and coworkers [16], as well as SM3 medium described by Nicaud et al. [4]. To select the best medium for hIFN α2b production by JMY1852p strain, these media as well as the SM4 medium (designed for heterologous production in *Pichia pastoris*) [17] were assessed in shake-flask cultures (Figure 1).

After 72 h of cultivation, culture supernatants were collected, concentrated and subjected to western blot analysis using a monoclonal antibody directed towards hIFN α2b; bands at approximately 19 kDa were detected in all media except in the chemically defined medium SM3 which was then considered as unsuitable for hIFN α2b production and was withdrawn from the study (Figure 1b).

The expression levels achieved in SM1 and SM2 media were very low compared to that observed in the complex medium (CM). No hIFN α2b production was detected in cell lysates from culture pellets of hIFN α2b producing strain in all tested media (data not shown). However, a small band below the predicted size of hIFN α2b protein was observed in the supernatants of the CM and SM4 media (Figure 1b) which may be in favor of degradation. To check this hypothesis we performed another western blot using a polyclonal antibody directed towards hIFN α2b. Two bands were detected at approximately 19 kDa and 14 kDa with a large amount for the second band in the case of SM1 and CM media but not for SM4 medium (Figure 1c). Hence, it seems that proteolysis affects significantly the target protein produced in these media.

Optical density (OD), pH and morphology changes during cultures were monitored to determine the effects of varying the culture medium. Table 1 shows data obtained for the different cultures. Comparing the chemical elemental composition of the different cultures media (Table 2) important differences can be observed. Nevertheless cell growth in SM1 medium showed a similar trend to that observed in SM2 medium, for the first 24 hours of culture. The maximal specific growth rate (μ) of the recombinant strain was equal to 0.18 h^-1 ^in SM1 and SM2 and both media exhibited a similar biological activity which was around 2-fold lower than the level obtained in CM medium (Table 2). The complex medium (CM) showed a faster growth (μ = 0.22 h^-1^), a maximal biomass level of 40.1 g/l and the highest amount of hIFN α2b. However, its performance remains still lower in terms of productivity compared to the SM4 medium (0.32 × 10^-2 ^mg hIFN α2b/g biomass vs 0.84 × 10^-2 ^mg/g) (Table 1).

**Table 1:** Growth characteristics of *Y. lipolytica *in the different media

	SM1	SM2	SM4	CM
				
μ^a ^(h^-1)^	0.18	0.18	0.16	0. 22
Maximal Biomass (g/l)	24.01	17.72	14.26	40.1
IFN yield (μg/l)	14	3	120	125
Y_( hIFN/X) _(mg/g)	0.58 × 10^-3^	0.17 × 10^-3^	0.84 × 10^-2^	0.32 × 10^-2^
Residual oleic acid (g/l)	1.4	0.7	0.54	1.1
Final pH	3.64	2.72	3.18	7.78
Cell morphology	Mycelium	yeast cells	yeast cells	yeast cells+
Biological activity (UI/mg)	0.42 × 10^7^	0.54 × 10^7^	0.97 × 10^7^	mycelium 1.03 × 10^7^
				

^*a*^μ; growth rate calculated from total biomass level (g/l).

**Table 2:** Composition of different growth media used in this study

*Components (g/l)*	*SM1*	*SM2*	*SM3*	*SM4*
K_2_HPO_4_	-	5.5	-	-
KH_2_PO_4_	1	15	2	-
K_2_SO_4_	-	-	-	18.2
MgSO_4_.7H_2_O	0.5	1	0.6	7.28
CaCl_2_	0.1	-	5	-
NaCl	0.1	-	-	-
CaSO_4_	-	-	-	0.93
KOH	-	-	-	4.4
(NH_4_)_2_SO_4_	-	4	4.5	-
Urea	2	-	-	-
Glucose	20	20	20	20
H_3_PO_4_	-	-	-	26.7
**Trace elements (mg/l)**				^a^
H_3_BO_3_	0.5	-	8	20
CuSO_4_.5H_2_O	0.04	-	100	6000
KI	0.1	-	0.9	80
FeCl_3_.4H_2_O	0.2	10	-	-
ZnSO_4_.7H_2_O	0.4	-	4000	-
MnSO_4_. 2H_2_O	0.4	-	6500	3000
EDTA	-	-	12540	-
NaH_2_PO_4_.H_2_O	-	-	3000	-
FeSO_4_.7H_2_O	-	-	2500	6500
Na_2_MoO_4_.2H_2_O	-	-	4	200
CoCl_2_.6H_2_O	-	-	7	500
NiSO_4_.7H_2_O	-	-	0.8	-
ZnCl_2_	-	-	-	20000
**Vitamins (μg/l)**			^b^	
Biotin	8	8		80
Thiamin	200	100		-
Myo-inositol	4	2		-

**pH**	4.5	6	5.5	5

^a ^Concentration in the stock solution. 2 ml/l stock solution were added to SM4 medium

^b ^For SM3 medium, the vitamin solution is composed of 1 mg/l pantothenic acid, 1 mg/l nicotinic acid, 25 mg/l myo-inositol, 1 mg/l thiamin and 1 mg/l pyridoxol hydrochloride, 0.2 mg/l p-amino benzoic acid and 0.05 mg/l biotin. 1.5 ml of this solution were added to 1l of SM3 medium.

#### Influence of nitrogen source on biomass and hIFN α2b production

One of the most important parameter in media formulation is the nitrogen source. Various mineral and nitrogen compounds were evaluated as an enhancing factor of *Y. lipolytica *growth and hIFN α2b production. We had particularly studied the substitution of urea by ammonium sulfate for SM1 medium and the enrichment of SM1 and SM2 media with casaminoacids, tryptone and yeast extract. SM1 and SM2 media without any supplement were used as control.

**Figure 2:**
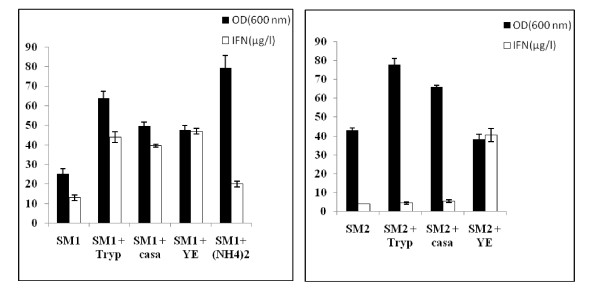
**Effect of nitrogen source on JMY1852p growth and hIFN α2b production**. The recombinant strain was grown in SM1 and SM2 media supplemented with tryptone (Tryp) at 10 g/l, casaminoacids (casa) at 5 g/l and yeast extract (YE) at 5 g/l, at 28°C and 180 rpm. (NH_4_)_2 _stands for (NH_4_)_2_SO_4_. Results are mean values of three independent experiments.

As shown in Figure 2, substitution of urea by ammonium sulfate in SM1 increased greatly the biomass level whereas a slight enhancement was noticed for hIFN α2b production. This result differs significantly from other studies, which showed a repression of the extracellular lipase production by ammonium salts in *Y. lipolytica *[6,18].Valuable improvements of hIFN α2b production and biomass level were observed upon addition of organic compounds to the basal salt medium SM1 compared to the control.

For the synthetic medium SM2, cultures containing tryptone and casamino acids showed a higher biomass level compared to the control containing inorganic nitrogen source. However neither tryptone nor casamino acids were able to generate high level of hIFN α2b production. Moreover, in contrast to cell growth, the production level of hIFN α2b was enhanced over yeast extract (YE) addition. In comparison to the control, nearby 10-fold increase was observed. These observations suggest that YE, a complex mixture of amino acids and peptides [19], provide an alternative source of vitamins and oligo-elements for *Y. lipolytica*. Since these media are prone to fluctuations and are referred as semi-defined media, their use is not considered as cost-effective and they are not recommended for large scale production of heterologous proteins [10].

#### The Invitrogen medium

The synthetic medium SM4 was formulated by Invitrogen Corporation (Carlsbad, CA, USA) to provide appropriate chemical and nutritional environments for *Pichia pastoris *growth. To our knowledge, there are no previous reports describing the use of this medium for the expression of proteins by other hosts especially by *Y. lipolytica*. Surprisingly, the expression level of the recombinant strain was dramatically improved when the strain was grown in SM4 medium; a strong band at the expected size was observed (Figure 1b). Signal densities quantification of hIFN α2b band using the Image-J software revealed that signal intensities of hIFN α2b were approximately 9 and 40-fold respectively higher than those obtained in SM1 and SM2 media and was similar to that reached in the CM medium. Table 1 shows that the yield Y_(hIFNα2b/X) _was the highest in this medium, it was 2.7-fold higher than CM. Furthermore, the biological activity of hIFN α2b in SM4 medium was the highest compared to other minimal media.

The SM4 appeared as the best medium allowing efficient hIFN α2b production in *Y. lipolytica*, by combining high productivity and activity with low level of proteolysis (Figures 1b & 1c). SM4 as a low-cost and defined medium is ideal for large scale production. This result emphasizes the importance of evaluating various cultivation media for the production of heterologous production.

#### Morphology and lipid accumulation

Depending on nutritional and environmental conditions *Y. lipolytica *grows in two distinct cellular forms (mycelia and yeast like) [2,3,20]. Morphological analysis of *Y. lipolytica *recombinant strain growing in different media revealed that in CM medium, the strain grew as mixture of yeast-like and short mycelial cells; yeast cells were predominant (about 80%) and pseudohyphe represented only 20%. In SM1 as well as SM4 media more than 90% of the cells remained in the yeast form. However higher amounts of mycelium was obtained in SM2 medium.

**Figure 3:**
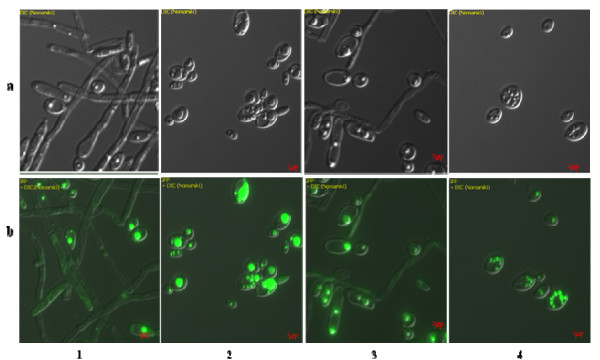
**Cell morphology of *Y. lipolytica *cultures in different media**. The recombinant strain was grown in SM2 (1), SM4 (2), CM (3) and SM1 (4) media. Depending on the culture medium yeast cell and/or mycelium were observed with interference contrast images (a). Micrographs were taken at 72 h using LipidTOX™ Green neutral lipid stains (b).

Conditions that induce the dimorphic transition are extremely variable. These include the presence of specific compounds in the culture media and pH levels; neutral or alkaline pHs induce filamentation while acid pH favors the yeast form [1]. It has been suggested that in *Y. lipolytica *pH affects the formation of hyphae indirectly by modulating the availability and/or utilization of transportable sources of nitrogen. Strains without functional alkaline extracellular proteinase (AEP), an enzyme providing transportable organic sources of carbon and nitrogen to cells growing on proteinaceous substrates, which is the case of our strain, did not respond to changes in pH in complex medium [20]. As shown in Figures 1 and 3, there is a relation between hIFN α2b production and cell morphology. hIFN α2b expression was observed only when the yeast form was predominant. This corroborates with data reported by Madzak et al. [8] who found that higher amounts of laccase activity expressed in *Y. lipolytica *were obtained in media that exhibit a lack of mycelium formation.

In addition, protein secretion in *Y. lipolytica *could be linked to dimorphism since several genes described in the secretion pathway are also implicated in morphological transition [8].

Besides morphology, cell size was notably affected by the amount of lipid bodies accumulated. As shown in Figure 3, lipid accumulation by the recombinant strain is medium dependent. Mlikova et al. [21] showed that structural changes on the surface of *Y. lipolytica *cells grown on oleic acid result in the formation of protrusions that enable the yeast to uptake the hydrophobic compounds from the medium. The uptake of oleic acid was very efficient in the SM4 medium compared to other media; large obese cells with discernible lipid bodies appeared in the cells grown in this medium. In this case, oleic acid was not only used as an inducer of hIFN α2b production but was also stored as lipid bodies. It is worth to mention that all media were not depleted of nitrogen.

Lipid storage in yeasts could be prevented using genetic engineering tools. Four yeast genes: *ARE1*, *ARE2, DGA1 *and *LRO1*, were found to contribute to triacylglycerol synthesis and lipid storage in *Saccharomyces cerevisiae *[22]. Sandager et al. [22] conducted series of genes disruption in *Saccharomyces cerevisiae*; they showed that the quadruple disrupted strain lost the capacity to accumulate lipids. They also demonstrated that neither lipid storage nor lipid bodies were essential for growth.

Further research, involving other strains and constructs, is needed to provide further insights about metabolic pathways of oleic acid in *Y. lipolytica *used for the production of heterologous proteins under the control of POX2 promoter.

### Adaptation of SM4 medium for optimal cultivation of Y. lipolytica

In the much-cited SM2 medium of Gordillo et al. [16], no oligo-elements except Fe^3+ ^were used. This indicates that Fe^3+ ^plays a key role in *Y. lipolytica *growth and metabolism. Furthermore, we showed that only the addition of YE, containing trace elements and vitamins at a concentration of 2 to 2000-fold higher than other organic sources, had increased significantly hIFN α2b production [23].

To further optimize SM4 medium composition, we assessed the effect of the following factors: FeCl_3, _vitamins solution enriched with myo-inositol and thiamin, and trace-elements solution on hIFN α2b expression. Nitrogen sources namely ammonium sulfate and glutamate were also evaluated. The L8 experimental Plackett-Burman matrix was applied to determine optimum conditions for hIFN α2b production by *Y. lipolytica *recombinant strain. The layout of the experiments carried out was given in Table 3.

**Table 3:** Experimental design for SM4 optimization using Modde 6.0 software

Exp No	Trace element	**FeCl**_**3 **_**(mg/l)**	Glutamate (g/l)	**(NH**_**4**_**)**_**2**_**SO**_**4 **_**(g/l)**	Vitamins	hIFNα2b level (μg/l)
1	PTM1	0	0	4	mixture	2.25
2	PTM1	10	0	0	biotin	135
3	PTM1	10	1	0	mixture	216
4	SM1	10	1	4	mixture	3.37
5	SM1	0	1	4	biotin	0.25
6	SM1	10	0	4	biotin	1.68
7	SM1	0	1	0	mixture	13.5
8	PTM1	0	0	0	biotin	121.5

*SM1: trace elements composition in the synthetic medium 1

**Figure 4:**
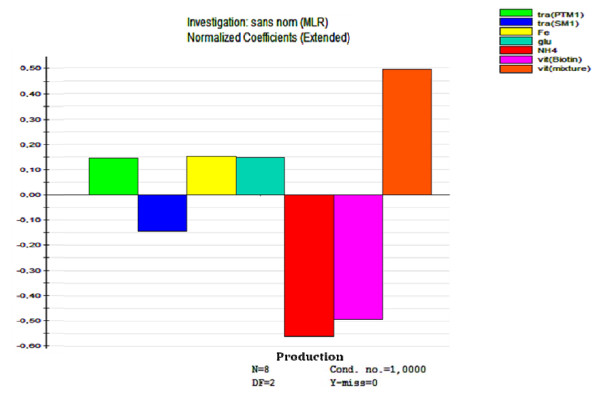
**Improvement of SM4 medium composition using an L8 Plackett-Burman design matrix**. Shake flask cultures were carried out with 25 ml of culture medium SM4 at 28°C and 180 rpm. FeCl_3, _vitamins solution enriched with myo-inositol and thiamin, trace-elements solution, ammonium sulfate and glutamate are used as described in Table 3. After Western blot analysis, the effect of each component on hIFN α2b production was analyzed by Modde 6.0 software. Results are mean values of three independent experiments.

Rates of growth were comparable for all media conditions tested (data not shown). As shown in Figure 4, among the factors studied, ammonium sulfate showed the most negative strong influence on hIFN α2b production. On the other hand, glutamate seemed to have a positive impact on the production. Glutamate is an essential precursor of protein and nucleotide synthesis. It is also an important substrate for energy metabolism. In addition glutamate was reported as a stimulating compound for aerobic glycolysis and acetylCoA carboxylase activation [24].

Addition of thiamin, a cofactor in the pyruvate dehydrogenase complex and the alpha ketoglutaric acid dehydrogenase [25,26], and myo-inositol as well as PTM1 solution (trace-elements solution, described in Table 2 for the SM4 medium) resulted in an increase of hIFN α2b yield. This suggests that vitamins and some trace elements are involved in the activity of basic enzymes responsible for oleic acid uptake and/or metabolism by *Y. lipolytica *cells. Our results correlate with those reported by Boze et al. [23] who found that supplementation of basal medium with seven vitamins and two trace elements enhanced the growth and recombinant protein production in *Pichia pastoris*.

On the other hand, our study revealed that FeCl_3 _has a positive effect on protein expression. Indeed, DNA microarray analysis of *S. cerevisiae *yeast cells grown under iron excess or iron starvation conditions reveals a decrease in mRNA levels for many metabolic pathways protein like mitochondrial respiration, heme and biotin biosynthesis in iron depleted cells [27,28]. Transcripts coding for iron-sulfur proteins involved in the synthesis of leucine, glutamate are also diminished.

The effect of olive oil, methyloleate, and oleic acid as inducer sources of hIFN α2b production in SM4 medium was evaluated. Contrary to other strains which was enhanced by olive oil and strongly inhibited by oleic acid [12], the production pattern was very similar for all the inducers tested. Slightly improvement was obtained with oleic acid (data not shown).

The highest hIFN α2b yield was achieved in the minimal medium detailed in the experiment N°3 (Table 3). Such a medium gave a hIFN α2b level that was around 2-fold higher than SM4. This medium composed of SM4 as a basal medium, 10 mg/l FeCl_3_, 1 g/l glutamate, 5 ml/l PTM1 solution and the mixture of vitamins was called GNY medium.

### Effect of PTM1 solution

Many types of yeast require for their proper propagation the presence of one or more micronutrient [11,29]. The effect of these elements on *Y. lipolytica *growth has not been widely investigated. In this study, the influence of PTM1 solution on cell growth and hIFN α2b production was assessed; two concentrations: 2 ml/l and 5 ml/l were tested and added to the basal salt medium SM4. In these experiments the recombinant strain was cultivated in a 5-l bioreactor in a batch mode, using oleic acid as a carbon source and inducer.

**Figure 5:**
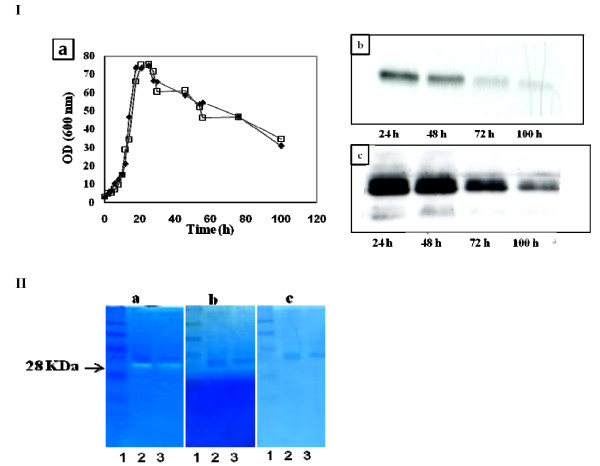
**Effect of PTM1 concentration**. (I-a) effect of PTM1 concentration on JMY1852p strain growth, hIFN α2b production using 2 ml/l of PTM1 solution (I-b) and 5 ml/l of PTM1 solution (I-c). Cultures were carried in 5-l bioreactor with oleic acid as carbon and inducer source, data represent the mean of duplicate. Open triangle: 5 ml PTM1 addition; closed triangle: 2 ml PTM1 addition. (II-a) a 28 kDa protease detected in cultures supernatants (SN). Lane 1; molecular weight, lane 2; SN (5 ml/l PTM1), lane 3; SN (2 ml/l PTM1). Protease inhibition by pepstatin addition at 10 μM (II-b) and 5 ml/l of PTM1 addition (II-c).

Growth rates were similar in the two culture conditions; maximal growth was reached at 24 h of culture followed by an immediate severe decrease of biomass. No stationary phase was observed (Figure 5Ia); this phenomenon could be explained by an excessive accumulation of lipid in cells that promotes cell lysis under high agitation. By contrast, the rate of hIFN α2b production was significantly enhanced at the highest concentration of PTM1. Figure 5Ib showed that the production was reduced to up 80% when PTM1 was supplemented to the culture medium at 2 ml/l especially, after 48 h of growth which correlates with the beginning of the decline phase and the release of proteases due to cell lysis compared to the medium containing 5 ml/l of PTM1 solution.

To investigate whether differences in hIFN α2b expression could be explained by protease degradation, supernatants from cultures were subjected to zymographic analysis. Several substrates, such as casein, gelatin and bovine serum albumin were used as substrate; a 28 kDa protease with casein specificity was identified at pH 5 in the two culture conditions. Colorimetric method with azocasein substrate, a chromogenic derivative of casein, showed no proteolytic activity at the start of cultures, appearance of proteases started at 20 h, their concentrations increased with increased cell concentration and at the end of cultures. A drastic increase of the target protein level was observed. Therefore, it seems likely that PTM1 solution appear to protect hIFN α2b against this protease (data not shown).

The effect of inhibitor supplementation on protease activity was studied; zymographies of samples showed no activity with pepstatin at 10 μM. Surprisingly complete inhibition of the proteolytic activity occurred upon addition of PTM1 at 5 ml/l in the enzymatic reaction (Figure 5 II). This result explains the low degradation in SM4 medium compared to data obtained in the other media (Figure 1c). The effect observed for PTM1 solution has not been reported previously which could be a solution to alleviate some proteolysis problems.

To further characterize the inhibition of hIFN α2b degradation by PTM1, the experimental Box-Behnkan design was applied, the effects of the eight components present in PTM1 solution (H_3_BO_3_; CuSO_4_.5H_2_O; KI; MnSO_4_.2H_2_O; FeSO_4_.7H_2_O; Na_2_MoO_4_.2H_2_O; CoCl_2_.6H_2_O and ZnCl_2_) and their interactions were investigated; 67 experiments were conducted according to the lay out detailed in Table 4. Proteolytic activity was measured by the colorimetric azocasein method. Experimental data were statistically analyzed by Modde 6.0 software to identify the effect of each factor and interaction studied.

**Table 4:** Lay out of the experiments conducted to study the effect of PTM1 components on protease activity.

Exp No	h3	co	zn	fe	k	mn	cu	na	
1	p	p	p	p	p	p	a	a	

2	a	p	p	p	p	p	p	p	

3	p	a	p	p	p	p	p	p	

4	a	a	p	p	p	p	a	a	

5	p	p	a	p	p	p	p	a	

6	a	p	a	p	p	p	a	p	

7	p	a	a	p	p	p	a	p	

8	a	a	a	p	p	p	p	a	

9	p	p	p	a	p	p	p	a	

10	a	p	p	a	p	p	a	p	

11	p	a	p	a	p	p	a	p	

12	a	a	p	a	p	p	p	a	

13	p	p	a	a	p	p	a	a	

14	a	p	a	a	p	p	p	p	

15	p	a	a	a	p	p	p	p	

16	a	a	a	a	p	p	a	a	

17	p	p	p	p	a	p	a	p	

18	a	p	p	p	a	p	p	a	

19	p	a	p	p	a	p	p	a	

20	a	a	p	p	a	p	a	p	

21	p	p	a	p	a	p	p	p	

22	a	p	a	p	a	p	a	a	

23	p	a	a	p	a	p	a	a	

24	a	a	a	p	a	p	p	p	

25	p	p	p	a	a	p	p	p	

26	a	p	p	a	a	p	a	a	

27	p	a	p	a	a	p	a	a	

28	a	a	p	a	a	p	p	p	

29	p	p	a	a	a	p	a	p	

30	a	p	a	a	a	p	p	a	

31	p	a	a	a	a	p	p	a	

32	a	a	a	a	a	p	a	p	

33	p	p	p	p	p	a	a	p	

34	a	p	p	p	p	a	p	a	

35	p	a	p	p	p	a	p	a	

36	a	a	p	p	p	a	a	p	

37	p	p	a	p	p	a	p	p	

38	a	p	a	p	p	a	a	a	

39	p	a	a	p	p	a	a	a	

40	a	a	a	p	p	a	p	p	

41	p	p	p	a	p	a	p	p	

42	a	p	p	a	p	a	a	a	

43	p	a	p	a	p	a	a	a	

44	a	a	p	a	p	a	p	p	

45	p	p	a	a	p	a	a	p	

46	a	p	a	a	p	a	p	a	

47	p	a	a	a	p	a	p	a	

48	a	a	a	a	p	a	a	p	

49	p	p	p	p	a	a	a	a	

50	a	p	p	p	a	a	p	p	

51	p	a	p	p	a	a	p	p	

52	a	a	p	p	a	a	a	a	

53	p	p	a	p	a	a	p	a	

54	a	p	a	p	a	a	a	p	

55	p	a	a	p	a	a	a	p	

56	a	a	a	p	a	a	p	a	

57	p	p	p	a	a	a	p	a	

58	a	p	p	a	a	a	a	p	

59	p	a	p	a	a	a	a	p	

60	a	a	p	a	a	a	p	a	

61	p	p	a	a	a	a	a	a	

62	a	p	a	a	a	a	p	p	

63	p	a	a	a	a	a	p	p	

64	a	a	a	a	a	a	a	a	

65	p	p	p	p	p	p	p	p	

66	p	p	p	p	p	p	p	p	

67	p	p	p	p	p	p	p	p	

The experiments were designed by Modde 6.0 software.

Abbreviations: h3 (H_3_BO_3_); cu (CuSO_4_.5H_2_O); k (KI); mn (MnSO_4_.2H_2_O); fe (FeSO_4_.7H_2_O); na (Na_2_MoO_4_.2H_2_O); co (CoCl_2_.6H_2_O) and zn (ZnCl_2_). (p) means presence and (a) stands for absence.

**Figure 6:**
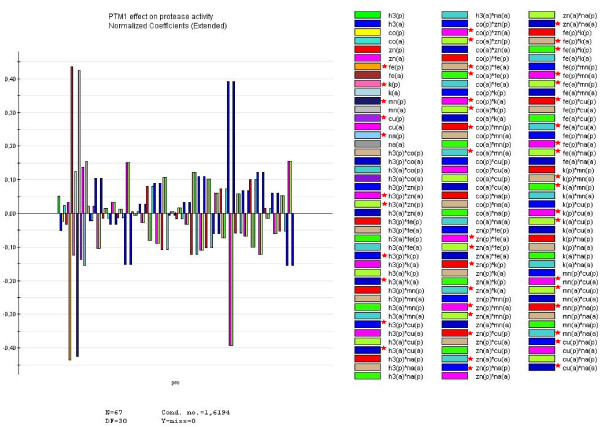
**Influence of mineral ions on the proteolytic activity (pro) (U/ml) of *Y. lipolytica***. H_3_BO_3_(h3); CuSO_4_.5H_2_O (cu); KI(k); MnSO_4_.2H_2_O (mn); FeSO_4_.7H_2_O (fe); Na_2_MoO_4_.2H_2_O (na); CoCl_2_.6H_2_O (co) and ZnCl_2 _(zn) were added at different combination to the colorimetric reaction for proteolysis activity determination using azocasein as substrate. (a) absence, (p) presence. Results are mean values of two independent repeat of 67 experiments. *indicates trace-elements and interactions that have an inhibitory effect on the proteolytic activity of *Y. lipolytica*

As shown in Figure 6, among all various trace elements tested, FeCl_3 _and MnSO_4 _have the most inhibitory effect on the proteases followed by KI, CuSO_4 _and Na_2_MoO_4_. Others elements like CoCl_2_, ZnCl_2 _have a less pronounced effect whereas H_3_BO_3 _acted in the opposite way. Furthermore, FeCl_3 _without MnSO_4 _or MnSO_4 _without FeCl_3_, showed an inhibition of the proteolysis. Similar data were observed for H_3_BO_3_xZnCl_2_, CoCl_2_xFeCl_3_, CoCl_2_xKI, ZnCl_2_xMnSO_4_, FeCl_3_xKI, KIxMnSO_4_, KIxCuSO_4, _MnSO_4_xCuSO_4 _and FeCl_3_x Na_2_MoO_4 _interactions. Other interactions showed a weaker inhibitory effect (Figure 6). These data indicate the positive effect of some mineral ions for the improvement of heterologous protein expression by the non conventional yeast *Y. lipolytica*.

Literature review shows that these ions could have an activating or inhibiting role in protein production [11,29]. Zhang et al. [29] reported that bivalent cations such as Mn^2+ ^and Mg^2+ ^increased the production of extrasucrase by *Escherichia coli *whereas Zn^2+^, Fe^2+ ^and Cu^2+ ^have an opposite effect. Nevertheless, the enhancement of protein production via proteolytic inhibition as described in this study has not been reported previously.

### Bioreactor culture in GNY medium

The effect of the new formulated medium GNY on hIFN α2b production by *Y. lipolytica *recombinant strain under the control of the inducible fatty acid promoter POX2 was investigated in a batch bioreactor culture, under controlled conditions as described in the material and methods section. Two phases cultivation was conducted, the first one is a cell growth with glucose as carbon source and the second one is the induction of hIFN α2b biosynthesis with oleic acid.

Kinetics of biomass and hIFN α2b expression over 120 h of culture on 2% oleic acid was monitored. After 24 h of culture on 20 g/l glucose, the biomass reached 20 g DW/l and 35 g/l once oleic acid was used as an inducer. Culture supernatants were analyzed by Western blot under reducing conditions, hIFN α2b production was initiated after 2 h of induction. Maximum yield of hIFN α2b was equal to 50 mg/l with a biological activity of 2.1 × 10^7^IU/mg.

Compared to shake flask procedure, culture in bioreactor did not permit to enhance significantly the biomass yield however it generates a drastically higher expression level. Over 416-fold increase of hIFN α2b concentration and 2-fold enhancement of the biological activity were obtained. This is the first study describing this amount of increase; only 8-20 fold increase in heterologous protein expression has been reported in *Y. lipolytica *when scaling-up cultures from shake flask to bioreactor [5,8]. However, the fold of increase of the biological activity of hIFN α2b observed was not proportional to the increase of the production level. Biological activity of hIFN α2b can be influenced by several factors such as the post-translational modifications; any factor that interferes or favors the binding of this cytokine to its receptor, impacts the bioactivity of hIFN α2b [30]. Therefore, further structural studies are needed to give deeper insights about this result.

## Conclusions

Conventional methods as well as statistical experimental designs were used in this study to select and optimize a minimal defined medium for *Y. lipolytica *heterologous protein expression. The optimized medium GNY is suitable for the production of hIFN α2b by *Y. lipolytica *JMY1852p with the advantage that no complex nitrogen sources with non-defined composition were required. Nutritional composition of the culture medium especially trace elements plays an important role in the improvement of protein production. GNY appears as an attractive medium for heterologous production in *Y. lipolytica*. Besides hIFN α2b production, the expression of other therapeutic proteins by this host cultivated in GNY medium is currently under investigation. Promising results could be expected from a more complete optimization strategy of growth and induction conditions in bioreactor.

## Materials and methods

### Microorganism

The auxotrophic, recombinant strain of *Y. lipolytica *Po1d (JMY 1852) with the genotype [*MATA, leu2-270, ura3-302, xpr2-322*, pox2^- ^preLip2-IFNop-*URA3*], producing heterologous hIFN α2b was constructed previously [13]. The synthetic gene optimized for its codon bias was expressed under the control of the POX2 promoter which is induced by oleic acid. In this study, a prototrophic derivative was obtained by transformation with a *SalI *fragment carrying the *LEU2 *gene. This latter prototrophic strain was called JMY 1852p and used throughout the present investigation.

### Chemicals

Chemicals were purchased from Sigma-Aldrich (St. Louis, MO, USA) except oleic acid which was provided by Prolab (Quebec, Canada). Thiamin was from Merck (Darmstadt, Germany) and Myo-inositol was from Calibiochem (La Jolla, Canada).

### Media

The recombinant strain was isolated on YPD-agar (Yeast Peptone Dextrose) medium (20 g/l glucose; 10 g/l yeast extract; 10 g/l peptone and 20 g/l agar) and was grown in complex rich medium Y_1_T_2_D_1_O_2 _or in defined media. The Y_1_T_2_D_1_O_2 _medium consisting of 10 g/l yeast extract, 20 g/l bactotryptone, 20 g/l glucose and 2% (W/V) oleic acid was buffered with 50 mM sodium phosphate buffer, pH = 6.8. The compositions of the four mineral media used in this study are described in Table 2.

Media pHs were adjusted at the required values with NaOH (5N) or ammonia prior to sterilization. The trace elements and vitamins solutions, sterilized by filtration, were added to the culture media as described in the text.

Oleic acid was added to these media to a final concentration of 20 g/l. Stock solution (20% oleic acid, 0.5% Tween 20) was subjected to sonication for 2 min with a BANDELIN SONOPLUS sonicator (Berlin, Germany) for emulsification purpose.

### Culture conditions

For all experiments, pre-inocula were grown on YPD medium. Cells in mid-exponential growth (16 h at 28°C and 180 rpm) were centrifuged, washed twice with 50 mM phosphate buffer, pH 6.8 and used to inoculate the culture at an initial optical density at 600 nm (OD 600 nm) of 0.4. All cultures were performed at least in duplicate.

Shake flask cultures were carried out in 250 ml baffled Erlenmeyer flasks with 25 ml of culture medium and incubated at 28°C at 180 rpm. Samples were taken at various time intervals to monitor cell growth and hIFN α2b level. For purification purpose, cultures were dispensed in 250 ml volumes into 2-l baffled shake flasks. To study the effect of organic nitrogen source, media were enriched with either 10 g/l of tryptone, or 5 g/l of yeast extract or 5 g/l casamino acids.

Cultures were carried out in a 5-l bioreactor (Infors, Switzerland) with working volume of 2 l. After sterilization at 121°C for 30 min, the medium was inoculated with 200 ml of pre-culture at an initial OD 600 nm culture of 0.3. Culture was performed at 28°C, aeration rate of 1.5 vvm and agitation speed of 600 rpm. Samples for the determination of the production and cell dry weight were withdrawn at 2 h interval. Oleic acid concentration was estimated by the colorimetric method based on a sulfo-phospho-vanillin reaction described by Frings and Dunn (1970) [31]. Biomass was monitored either by measuring optical density (OD_600_) or by dry weight (DW) determination. One unit of OD was found to be equivalent to 0.3 g/l DW. When cells were grown on media containing oleic acid, samples were extracted with 2/5 (V/V) of propanol/butanol solution prior to optical density determination.

### Determination of protease activity

#### Colorimetric method

Protease activity was determined by the colometric method using azocasein as a substrate. Culture supernatant (10 μl ) was mixed with 10 μl of a 2.5% azocasein solution and 70 μl of 0.1 M phosphate-citrate buffer pH 5 then incubated at 28°C for 1 h. The reaction was stopped by addition of 350 μl of 10% TCA (Trichloro acetic acid) solution. Samples were centrifuged at 13.000 rpm for 10 min, and then the absorbances of the supernatant were read at 440 nm against the blank. One unit of protease activity was defined as the amount of enzyme required for an increase in absorbance by 0.01 per hour.

#### Zymographies

For zymographic analysis, 15% separating gels were mixed with 5 mg casein; samples were treated with three-fold concentrated sample buffer. Gels were run at constant current of 100 mv Afterwards the gels were rinsed three time with 2.5% (V/V) Triton X-100 and incubated overnight in 50 mM acetate buffer pH 5 with or without inhibitors or metals (CaCl_2 _5 mM, ZnCl_2 _1 μM, PTM1) [32]. Gels were stained with coomassie blue and then destained until transparent zones caused by proteolytic digestion of the protein substrate in the gel, are visible against a blue background.

### SDS-PAGE and Western blot analysis

Sodium dodecyl sulfate polyacrylamide gel electrophoresis (SDS-PAGE) was performed in 15% polyacrylamide gels under denaturating conditions as described by Laemmli [33] Proteins secreted into the medium and collected after 72 h of culture, were concentrated by microcon (Millipore, Bedford, MA, USA). After separation, proteins were stained with coomassie brilliant blue R 250.

Prestained broad range protein marker RPN756 (GE Healthcare, Uppsala, Sweden) was utilized for estimation of proteins molecular sizes.

For western blot analysis, 10 μl of concentrated supernatant were separated by SDS-PAGE and transferred onto nitrocellulose membranes (Millipore, Bedford, MA, USA) by electroblotting. The membranes were blocked with PBS-5% skimmed milk, 0.1% Tween 20 overnight at 4°C. Membranes were incubated for 1 hour with anti-human IFN-α monoclonal antibody produced in-house and diluted to 1/500 followed by incubation with a goat anti-mouse IgG peroxidase conjugated monoclonal antibody (Sigma, St Louis, USA) diluted at 1/5000 or with polyclonal antibody also produced in-house and diluted to 1/200. The immunoreactive protein was visualized by ECL (GE Healthcare, Uppsala, Sweden). Western blots were scanned and analyzed by J-image software (Image-J 1.42) to determine the area of each band. hIFN α2b was quantified using a calibration curve established with purified hIFN-α2b produced in *Pichia pastoris*.

### Fluorescence microscopy

To visualize lipid bodies, LipidTOX™ Green neutral lipid stains (2.5 mg/ml in ethanol from Invitrogen) was added to a cell suspension that has an OD 600 of 5. Microscopy was performed with a fluorescence microscope AXIO Imager.M1 (Zeiss, Le Pecq, France) at 495 nm with a 100 × oil immersion objective. AxioVision Rel. 4.6 software was used for recording the images.

### Biological activity

The biological activity of the hIFN α2b preparation was determined by gene report assay as described by Meager [34]. Briefly HEK (Human Embryo Kidney) 293P cell line stably transfected with IFN-inducible promoter sequence (ISRE, Interferon Stimulated Response Element) linked to SEAP gene (secreted alkaline phosphatase) and exposed to human IFNα increase expression of the reporter gene product in direct relation to the dose of human IFNα. The readout is a measure of this product's enzymatic action. hIFN α2b reference standard (code: 95/566) was kindly provided by Dr Meager (NIBSC, United Kingdom).

### Experimental design

The software Modde 6.0 (Umetrics, Sweden) was used in this study for the design of the experiments and statistical analysis of the data. This approach was previously applied for other optimization studies in our laboratory [35].

To identify the significant factors that affect the response (hIFN α2b yield, proteolytic activity), normalized coefficients are calculated by the software. To make the coefficients comparable when responses have different ranges, the coefficients are normalized, that is the coefficients are divided by the standard deviation of their respective response. The overview plot displays the coefficients values for the response as bar graphs. This plot shows therefore how the factors affect the response. Coefficient values higher than zero indicate that factor/interaction studied has a positive effect on the response; the highest the coefficient, the more important is the contribution of factor/interaction studied. On the opposite negative values show that the response is impaired by the factor/interaction studied

## Declaration of competing interests

The authors declare that they have no competing interests.

## Authors' contributions

NG carried out the experiments, performed the statistical design and drafted the manuscript. AA participated in bioreactor cultures and biological activity tests. JMN supervised clones design and selection and reviewed the manuscript. HK conceived the study, coordinated he experiments and reviewed the final manuscript. All authors read and approved the final manuscript.
